# High-Quality Genome Assembly of the White King Pigeon for Genetic Reference of Chinese Market Pigeon Breeds

**DOI:** 10.1038/s41597-026-07220-4

**Published:** 2026-06-18

**Authors:** Guang Yang, Tao Zeng, Lizhi Lu, Wenwu Xu, Chunqin Wu, Haiying Li, Yong Tian, Tiantian Gu, Li Chen, Yibo Zong, Yuguang Chang, Liyan Dong

**Affiliations:** 1 Wenzhou Vocational College of Science and Technology, Wenzhou, China; 2 Key Laboratory of Livestock and Poultry Resources (Poultry) Evaluation and Utilization, Ministry of Agriculture and Rural Affairs of China, Hangzhou, China; 3 Xinjiang Agricultural University, Urumqi, China; 4 State Key Laboratory for Managing Biotic and Chemical Threats to the Quality and Safety of Agro-Products, Institute of Animal Husbandry and Veterinary Science, Zhejiang Academy of Agricultural Sciences, Hangzhou, China; 5 Poultry seed industry and green breeding technology Zhejiang Engineering Research Center, Hangzhou, China

**Keywords:** Structural variation, Animal breeding

## Abstract

This study was conducted to generate a high-quality chromosome-level genome assembly of a female White King pigeon using a combination of Illumina short pair-end reads; HiFi reads and Hi-C technologies. Using the reference genome,Over 22,685,232 single nucleotide polymorphisms (SNPs) and 2,466,122 insertion-deletions (InDels) were identified. The genome sequence with a total length of 1.09 Gb was located on 29 chromosomes, accounting for 98.59%, while the number of corresponding sequences was 1,270, accounting for 61.53%. Among the sequences located on chromosomes, the length of the sequences that can determine the order and direction was 1.06 Gb, accounting for 96.8% of the total length of the sequences located on chromosomes. Sixty-four pigeons from six breeds of Chinese mainstream market were investigated and approximately 94.67% high-quality sequences(Q30 > 90%), with a mapping rate over 99.12% for each individual were observed, with a genome coverage of 99.12%. further contributing to the public database. This dataset provides valuable resources for studying genetic diversity and adaptation for the cultivation of new pigeon breeds.

## Background & Summary

Pigeons were domesticated approximately 5000 years ago in the Neolithic^1,2^. Their phenotype exhibits remarkable diversity, such as theatrical alterations in craniofacial formation, colors of plumage pigmentation, feather position and structure, vocalizations, and flying capability^3^, In 19th century Charles Darwin was fascinated by the rich diversity of pigeon, In his famous book Origin of species. Charles Darwin describes different pigeons to prove his point on evolution^4^. Now this study, we identified over 22.7 million SNPs and 2.5 million InDels using the assembled White King pigeon reference genome. In comparison with the White King pigeon genome, the diverse Chinese market pigeon breeds featured in this study display substantial genetic diversity at the molecular level.

Domestic pigeons (Columba livia) exhibit extraordinary phenotypic diversity resulting from long-term artificial selection, making them a classic model for studying genetic mechanisms underlying trait variation. Previous reviews have highlighted that pigeon breeds derive from a single ancestral species (the rock pigeon) and have identified core genes associated with derived traits such as head crests (EphB2), feathered feet (Pitx1, Tbx5), and plumage color (Tyrp1, Sox10)^5^. These studies emphasize the importance of high-quality reference genomes for dissecting the genetic basis of phenotypic diversity.

Pigeons were traditionally kept for emotional, religious, and cultural reasons but were mainly used for communication purposes^6^. When fresh meat was in short supply, many households used pigeons as a source of meat^7^. Egypt was the first country globally to domesticate and breed pigeons and consider their meat a delicacy^8^. With the continuous development of breeding technology, the United States announced the creation of the world’s first improved meat pigeon in the 1980s, the American King pigeon, which was an important milestone in the global meat pigeon industry and promoted the rapid development of pigeon breeding^9^. The pigeon breeding industry has progressed successively in many countries, including France, Spain, China, Britain, India, and Bangladesh^10^. Although pigeon production may never increase enough to compete with commercial poultry as a major food source, it is a significant addition to the diet and a source of substantial income.

Pigeons have been reared in China for a long time; however, most are not used for meat. In the 1980s, some breeds of pigeons with excellent performance were introduced to China from abroad, and the large-scale pigeon breeding industry began to develop^10^. After 40 years of development, China has become the largest producer of pigeon meat worldwide, with an annual production of approximately 680 million squabs, accounting for approximately 80% of the global production^11^. Currently, the Chinese meat pigeon market relies on imported varieties: the Silver King Pigeon (America), White King pigeon (America), and Carnean pigeon (France). However, in individual provinces, local Chinese pigeons have dominated: the Shack Kee pigeon (Guangdong) and Tarim Pigeon (Xinjiang)^12^. The Pingyang pigeon was confirmed by the Ministry of Agriculture and Rural Affairs of China as a new variety in 2023 (Ministry of Agriculture and Rural Affairs Announcement No. 762 http://www.moa.gov.cn/govpublic/nybzzj1/202403/t20240313_6451328.htm). Therefore, it has the potential to dominate the Zhejiang pigeon meat market.

**Fig. 1:**
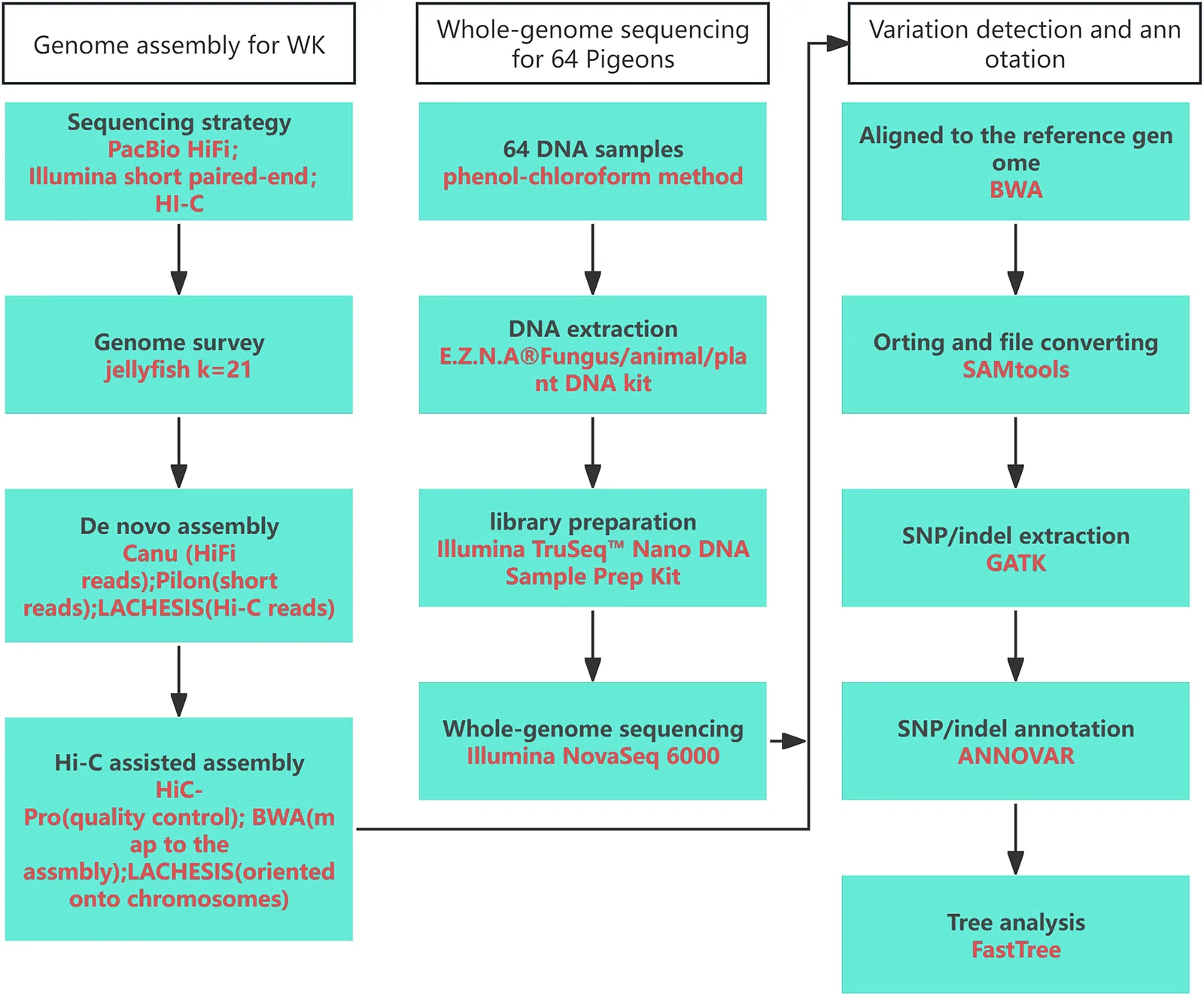
The workflow from sampling to genome and variant filtration.

This study provides a whole-genome sequencing dataset of 64 pigeons from the Chinese mainstream market from 6 local pigeon meat breeds, and a chromosome-level genome of the White King pigeon (WK) was assembled by combining Illumina short pair-end reads,Pacbio HiFi (high fidelity) reads, and Hi-C (High-throughput chromosome conformation capture) sequencing data. We extracted genomic DNA from blood, constructed different libraries, and sequenced them using an appropriate platform. After quality filtering and trimming of the raw data, we used Canu^13^ and Pilon^14^ to assemble and polish, the genome. Further, using the assembled White King pigeon genome, we identified over 22,685,232 SNPs and 2,466,122 InDels. This database represents an important supplement to the pigeon variant database and plays a crucial role in reconstructing the demographic and domestication history of pigeons. The WK genome assembled in this study provides a valuable genetic resource for future efforts to study pigeons and further comparative analysis of genome biology among pigeon species to promote breeding research. The workflow from sampling to genome and variant filtration is shown in Fig. 1.

## Methods

### Sampling

Blood samples from 64 adult pigeons of the following breeds were collected in Zhejiang Province, Xinjiang Province, and Guangdong Province for whole-genome sequencing: Silver King Pigeon (EK), White King pigeon (WK), Carnean pigeon (RC), Shack Kee pigeon (SK), Tarim Pigeon (TR), and Pingyang Pigeon (PY) (Supplement Table S1 Supplement information for 6 pigeon breeds). We collected a 1-ml blood sample from the wing vein of each individual and stored it in a 2 ml anticoagulation tube at −20 °C. A 244 day female WK was used for genome sequencing and assemb. The genotype of the female pigeon is ZW Containing more chromosomes.All procedures associated with the pigeon used in this study were consistent with the standards of the Laboratory Animal Guidelines for the Ethical Review of Animal Welfare and were approved by the Animal Use Committee of Zhejiang Academy of Agricultural Sciences (No. 2022ZAASLA68).

### Genome sequencing

#### Library construction and sequencing for whole-genome resequencing

Genomic DNA was extracted from each individual pigeon(Six varieties) using the E.Z.N.A. Tissue DNA kit (Omega Bio-Tek) following the Tissue DNA-Spin Protocol. After the DNA samples were delivered, a quality control test was conducted on the specimens, and the qualified DNA (>3 µg; concentration >30 ng/µl; OD260 /OD280 = 1.80~2.00) was used for further analysis. We used Illumina Hiseq X pair-end sequencing (PE150 mode) to conduct resequencing. For Illumina pair-end sequencing, at least 3 μg genomic DNA was used for sequencing library construction for each sample. Paired-end libraries with insert sizes of ~450 bp were prepared according to Illumina’s standard genomic DNA library preparation procedures. Purified genomic DNA was sheared into smaller fragments of the desired size using Covaris, and T4 DNA polymerase was used to generate blunt ends. After adding an ‘A’ base to the 3′ end of the blunt phosphorylated DNA fragments, adapters were ligated to the ends of the DNA fragments. The desired fragments were purified using gel electrophoresis, and then selectively enriched and amplified using polymerase chain reaction (PCR). The index tag was introduced into the adapter at the PCR stage as appropriate, followed by a library quality test. Finally, the quantified Illumina paired-end library was used for Illumina NovaSeq6000 sequencing at BIOZERON Co., Ltd (150 bp*2, Shanghai, China).

#### library construction and sequencing for de novo whole genome assembly and annotation

Genomic DNA was extracted from the blood(WK) using the standard phenol-chloroform extraction method for DNA sequencing library construction. The experimental procedures were performed according to the standard protocol provided by Illumina, and two types of libraries were constructed. Consistent with the *de novo* assembly workflow, genomic sequencing relied on two complementary platforms: the Illumina NovaSeq6000 platform generated paired-end reads for genome surveying and polishing of the initial assembly, while the PacBio Sequel II platform—operated in CCS mode—produced HiFi genomic reads to support high-continuity contig construction. For the Illumina platform, a short-read paired-end sequencing library with an insert size of 350 bp was prepared according to the manufacturer’s protocol, and sequenced on an Illumina NovaSeq6000 platform at Biomarker Technologies Co., Ltd. (150 bp*2, Beijing, China). This resulted in accurate short reads of 66.40 Gb (read length 350 bp, representing ~56.2× sequencing depth). The second libraries were sequenced using the PacBio Sequel II platform (CCS mode) in Biomarker Technologies Co., Ltd. (Beijing, China). We obtained 164.59 Gb of clean PacBio HiFi reads (average subread length of 12.13 kb), achieving a sequencing depth of ~148.59× (total base pairs of clean reads divided by the estimated 1.18 Gb genome size).

#### Hi-C library preparation and sequencing

The fixed cells(Peripheral blood lymphocytes in the blood of WK) were resuspended in 1 ml of lysis buffer (10 mM Tris-HCl pH 8.0, 10 mM NaCl, 0.2% IGEPAL CA-630, 1/10 vol) of proteinase inhibitor cocktail (Sigma), and then incubated on ice for 20 min. Nuclei were pelleted using centrifugation at 4 °C, 600 × g for 5 minutes, and then washed with 1 ml of the lysis buffer, followed by another round of centrifugation under similar conditions. After washing twice with restriction enzyme buffer, the nuclei were resuspended in 400 μl of restriction enzyme buffer and transferred to a safe-lock tube. Next, the chromatin was solubilized with dilute SDS (Sodium dodecyl sulfate) and incubation at 65 °C for 10 min. After Quenching the SDS by Triton X-100, it was digested with a 4 cutter restriction enzyme (400 units MboI) at 37 °C on a rocking platform. The next steps were Hi-C-specific, including marking the DNA ends with biotin-14-dCTP and performing blunt-end ligation of the cross-linked fragments. Proximal chromatin DNA was ligated using a ligation enzyme. The nuclear complexes were reversed crosslinked by incubating with proteinase K at 65 °C. DNA was purified using phenol-chloroform extraction. Biotin-C was removed from non-ligated fragment ends using T4 DNA polymerase. The fragments were sheared to 200–600 bp by sonication. Fragment ends were repaired using a mixture of T4 DNA polymerase, T4 polynucleotide kinase, and Klenow DNA polymerase. Biotin-labelled Hi-C samples were enriched using streptavidin C1 magnetic beads. A-tailing was added to the fragment ends by Klenow(exo-), and then an Illumina paired-end sequencing adapter was added by the ligation mix. Finally, Hi-C libraries were amplified by 12–14 cycles of PCR and sequenced on an Illumina HiSeq platform. Sequencing interaction patterns were obtained using an Illumina HiSeq instrument with a 2 × 150 bp read. Ultimately, we obtaineda total of 170,746,731 pairs of uniquely matched reads were obtained from the library, of which 39,784,599 were valid Hi-C data, accounting for 23.3% of uniquely matched genomic data.

#### Survey for pigeon genome

**Fig. 2:**
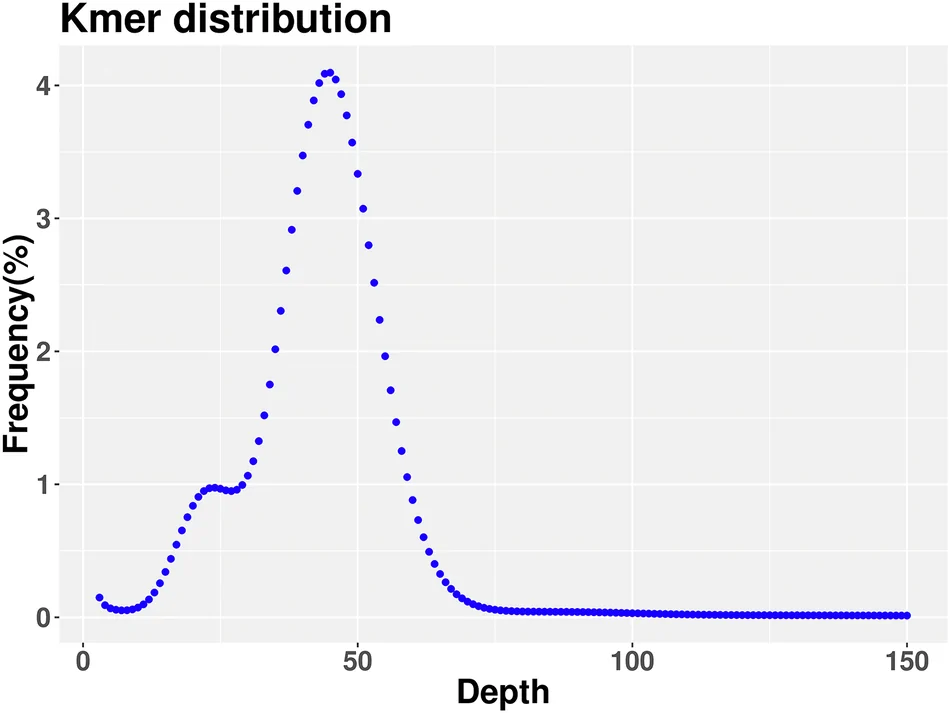
K-mer(K = 21) distribution.

To understand the genomic characteristics of pigeons, k-mer analysis using Illumina short paired-end reads was performed to estimate the genome size, heterozygosity, and repetitive sequences before genome assembly. Briefly, the genome size of the pigeons was estimated by counting k-mers with a length of 21; these k-mer frequencies fit a Poisson distribution (Fig. 2). A total of 57,469,444,515 reads were obtained, and 51,989,298,803 reads were used for K-mer analysis after filtering for quality. After calculation, the genome size was 1.18 Gbp (G = K-num/K-depth); the heterozygosity was estimated to be 0.59% and the repetitive sequence content was estimated to be 14.76%. K-mer counting was performed using Jellyfish^15^.

#### De novo assembly of the White King Pigeon genome

The *de novo* assembly of contigs was performed using Canu^13^, with parameter settings in which reads longer than 1,500 bp were selected for error correction with a parameter-corrected ErrorRate of 0.040. High-quality PacBio subreads were then used for genome assembly using Canu^13^. The resulting assembly was polished with Pilon^14^ using Illumina short paired-end reads. Three rounds of Pilon were performed using the default settings to fix bases, fill gaps, and correct local misassemblies until convergence, with minimal differences. We assessed the completeness of the assembly by recovering gene counts using Benchmarking Universal Single-Copy Orthologs (BUSCO)^16^. The BUSCO results are shown in Table 1. Hi-C reads were aligned to the assembly scaffolds using LACHESIS^17^. Finally, we assembled the White King Pigeon genome using 13,571,306 PacBio reads (average subread length of 12.13 kb and total length 164.59 Gb, representing ~148.59 × sequencing coverage) and Illumina short paired-end reads (read length 350 bp and total length 66.40 Gb, representing ~56.2 × sequencing coverage). After polishing, we obtained an assembly that consisted of 2,053 contigs with an N50 of 7.49 Mb (i.e., 50% of the assembly was contained in contigs ≥7.49 Mb in size) and a total length of 1.11 Gb.We designated this high-quality White King Pigeon genome assembly as ZA_WK_1. A detailed comparison of ZA_WK_1 with the previously reported pigeon genome assembly (ASM2865442v1) is provided in Table 2.

**Table 1:** BUSCO assessment results.

Type	Number	Percent(%)
Complete BUSCOs(C)	4595	93.49
Complete and single-copy BUSCOs(S)	4538	93.49
Complete and duplicated BUSCOs(D)	57	1.16
Fragmented BUSCOs(F)	205	4.17
Missing BUSCOs(M)	115	2.34
Total Lineage BUSCOs	4,915	100

**Table 2:** Comparison of Assembly Statistics Between ZA_WK_1 and ASM2865442v1.

Assembly name	Contig number	Contig length (bp)	N50 (bp)	L50 (bp)	GC content (%)	Gap total Length (bp)
ZA_WK_1	2,053	1,107,651,708	7,491,769	32	41.9	0
ASM2865442v1	1949	1176706617	7787539	44	42.5	22200

#### Hi-C assisted genome assembly

**Fig. 3:**
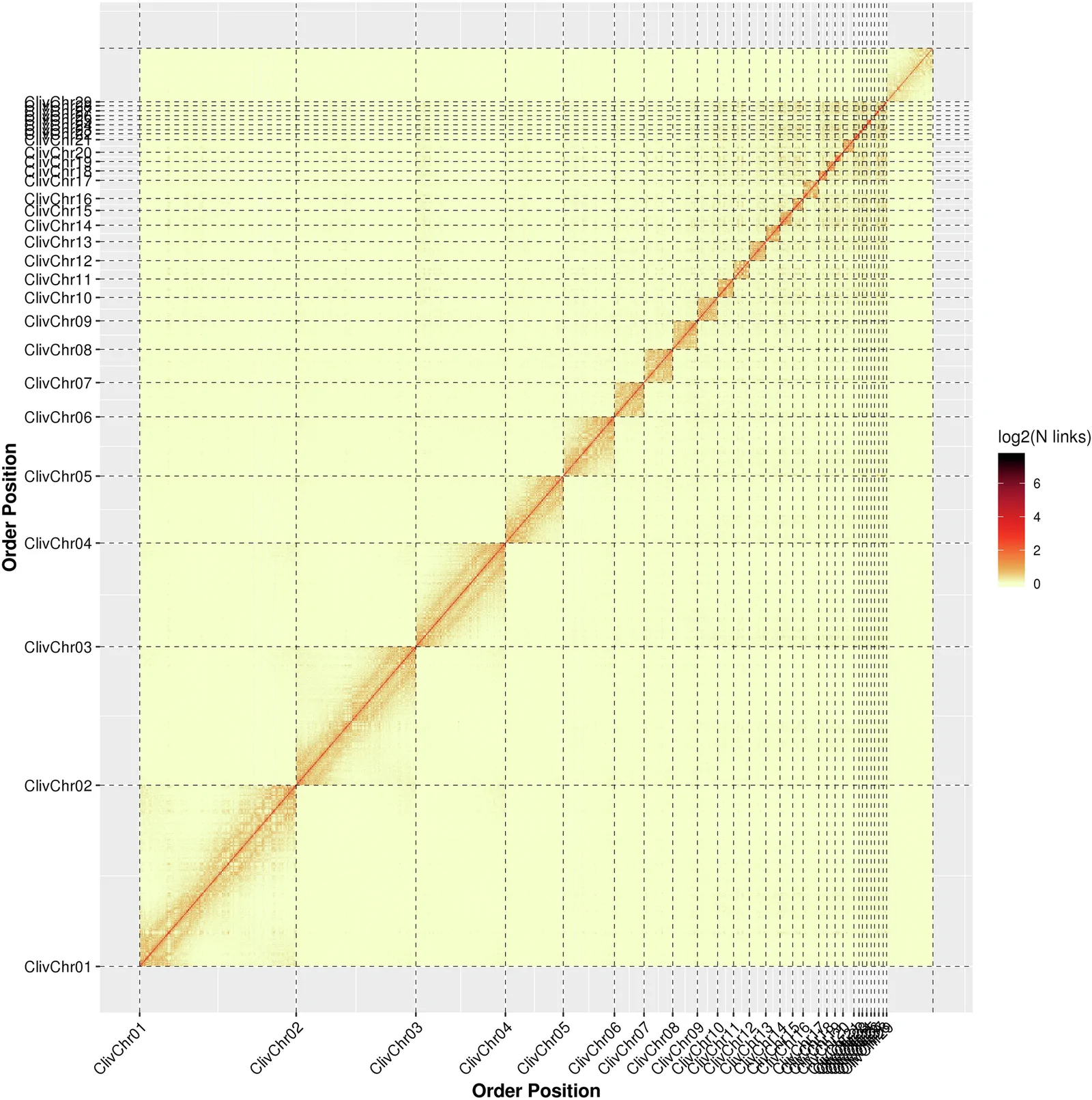
Genome-wide all-by-all Hi-C interation.

Quality control of the Hi-C raw data was carried out with HiC-Pro^18^ software. First, low-quality sequences (quality scores < 20), adaptor sequences, and sequences shorter than 30 bp were filtered out using fastp. Second, the clean paired-end reads were mapped to the assembly using BWA (Table 3)^19^. Third, valid interaction paired reads were identified from unambiguously mapped paired-end reads and retained by HiC-Pro for further analysis. HiC-Pro filters out invalid read pairs, such as dangling ends, self-cycles, re-ligations, and dumped products. The scaffolds were clustered, ordered, and oriented onto chromosomes using LACHESIS^17^. Finally after Hi-C assembly and manual adjustment by Juicebox^20^, the genome sequence with a total length of 1.09 Gb was located on 29 chromosomes, accounting for 98.59%, while the number of corresponding sequences was 1,270, accounting for 61.53%. Among the sequences located on chromosomes, the length of the sequences that could determine the order and direction was 1.06 Gb, accounting for 96.8% of the total length of sequences located on chromosomes.ZA_WK_1 meets all the core criteria for a chromosome-scale genome assembly, as defined in top genomics journals (e.g., Genome Biology, Nature Communications): (1) 98.59% of the total assembled genome length (1.07 Gb out of 1.09 Gb) is anchored to 29 chromosomes, exceeding the ≥90% threshold; (2) 96.8% of the chromosome-anchored sequences (1.06 Gb) have unambiguous order and orientation, satisfying the ≥90% requirement; (3) The scaffold N50 reaches 77.02 Mb, well above the ≥50 Mb standard for chromosome-scale assemblies.Consequently, the final chromosome-level genome assembly of ZA_WK_1 spans 1.06 Gb (representing the length of sequences with resolved order and orientation), with a contig N50 of 7.49 Mb and a scaffold N50 of 77.02 Mb (Table 4). A Hi-C interaction heatmap was generated to visualize intrachromosomal and interchromosomal interactions, further validating the accuracy of chromosome clustering and ordering (Fig. 3).

**Table 3:** Genomic statistics after Hi-C assembly.

Scaffold number	Scaffold length (bp)	Scaffold N50 (bp)	Scaffold N90 (bp)	Scaffold max (bp)	Gap total length (bp)
1,439	1,107,714,208	77,020,822	10,851,550	208,435,198	62,500
Contig number	Contig length (bp)	Contig N50 (bp)	Contig N90 (bp)	Contig max (bp)	GC content (%)
2,064	1,107,651,708	7,491,769	315,988	70,194,844	41.91

**Table 4:** Hi-C assembly data statistics.

Group	Cluster Num	Cluster Len	Order Num	Order Len
ClivChr01	73	210,758,247	43	208,430,998
ClivChr02	44	160,413,557	31	159,350,716
ClivChr03	38	120,363,466	24	119,251,430
ClivChr04	21	77,686,960	14	77,019,522
ClivChr05	12	68,487,585	10	68,195,960
ClivChr06	20	40,242,394	11	39,442,019
ClivChr07	14	38,571,518	10	38,236,423
ClivChr08	13	33,170,393	9	32,887,688
ClivChr09	8	26,996,886	6	26,921,390
ClivChr10	14	21,509,029	8	21,271,060
ClivChr11	5	21,110,470	5	21,110,470
ClivChr12	11	22,075,402	8	21,905,681
ClivChr13	24	19,359,959	15	18,831,002
ClivChr14	15	17,190,444	12	17,049,895
ClivChr15	16	14,214,185	11	13,812,173
ClivChr16	12	21,292,049	6	20,866,129
ClivChr17	16	11,183,152	11	10,980,011
ClivChr18	28	11,660,993	15	10,850,150
ClivChr19	14	10,904,214	11	10,618,879
ClivChr20	16	15,035,928	9	14,568,813
ClivChr21	14	7,324,126	7	6,819,378
ClivChr22	19	5,056,215	7	4,514,891
ClivChr23	11	5,906,193	7	5,820,977
ClivChr24	11	6,128,517	5	5,896,524
ClivChr25	205	12,762,962	36	4,664,921
ClivChr26	20	6,360,885	8	5,722,326
ClivChr27	32	7,291,838	16	5,860,418
ClivChr28	30	6,218,042	11	4,656,897
ClivChr29	514	72,797,948	288	61,615,428
Total(Ratio %)	1270(61.53)	1092073557(98.59)	654(51.5)	1057172169(96.8)

#### Variation detection and annotation

The raw paired-end reads were trimmed and quality controlled using Trimmomatic^21^ with parameters, and the clean sequencing reads were aligned to the reference genome sequence using BWA^19^ software. Sequence Alignment Map (SAM) format files were imported into SAMtools^22^ for sorting and merging and into Picard to remove duplicated reads. Sequencing depth and coverage were calculated based on the alignments using custom Perl scripts. The valid BAM file was used to detect SNPs and short InDel using the GATK “HaplotypeCaller” function (http://www.broadinstitute.org/gatk/). Variant call format (VCF) files were generated via quality filtering using GATK (VariantFiltration). When the gene annotation file of the reference genome is provided, annotation of the detected variations can be performed using ANNOVAR (http://www.openbioinformatics.org/annovar/), including SNP (synonymous or non-synonymous mutations of SNPs) and InDels. Structural variations (SV) were identified using BreakDancer^23^ (http://breakdancer.sourceforge.net).

#### Tree analysis

**Fig. 4:**
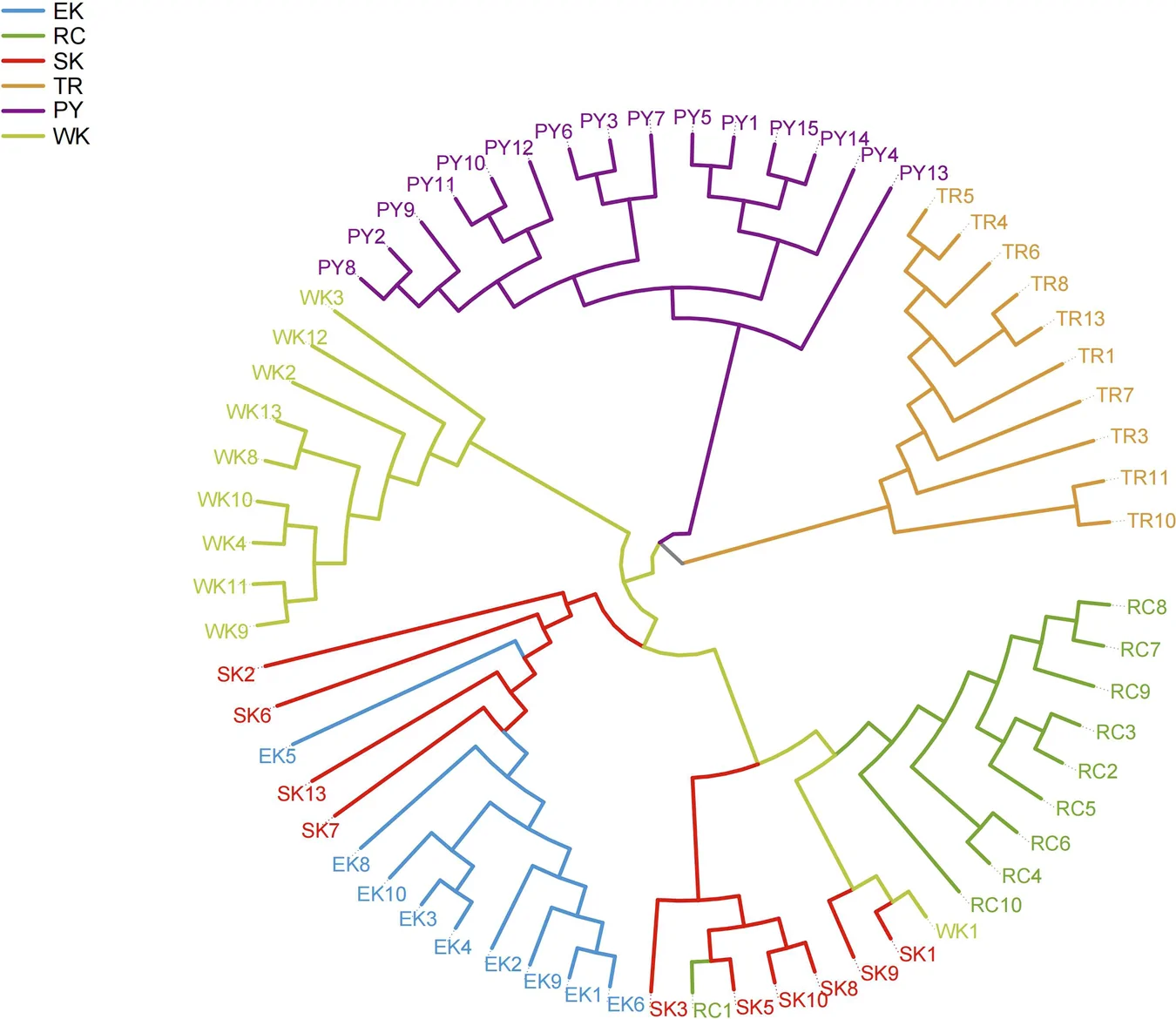
Neighbor-joining phylogenetic tree of 6 breed pigeon.

A neighbor-joining phylogenetic tree was constructed using the SNP data, an in house script to compute sample distance, and FastTree^24^ to construct the NJ (Neighbor-Joining) tree (Fig. 4).

#### Repeat sequence annotation

First, we used LTR_FINDER^25^ to predict full-length long terminal repeat (LTR) retrotransposons and *de novo* identification of repeat families in Silver King Pigeon genomes using RepeatScout^26^. Thus, we built a library of repeats of the Silver King Pigeon genome. Second, we used PASTEClassifier^27^ to classify Transposable elements (TEs) for the library. Then, the obtained library was aligned with TEclass Repbase (http://www.girinst.org/repbase)^28^ to classify the type of every repeat family. To identify repeats across the genome, RepeatMasker^29^ was used to search for both known and novel TEs by mapping sequences against the *de novo* repeat and Repbase TE libraries. The specific results are shown in Table 5.

**Table 5:** Repeat sequence statistics.

Type		Number	Length	Rate(%)
Class I	Total	401,663	110,471,440	9.97
	DIRS	1,416	440,966	0.04
	LTR(Copia)	1,014	423,754	0.04
	LTR(Gypsy)	4,745	391,688	0.04
	LTR(Unknown)	43,788	21,767,558	1.97
	PLE	3,467	2,043,055	0.18
	SINE	1,220	165,826	0.01
	TRIM	247	105,433	0.01
	Unknown	390	35,495	0.00
	Total	401,663	110,471,440	9.97
	Crypton	417	26,720	0.00
Class II	Helitron	1,909	296,318	0.03
	MITE	24	4,175	0.00
	Maverick	999	94,579	0.01
	TIR	19,550	2,743,894	0.25
	Unknown	8,756	685,745	0.06
	Total	31,655	3,663,280	0.33
Potential Host Gene		199	90,603	0.01
SSR		1,281	1,254,655	0.11
Unknown		7,049	1,915,250	0.17
Total		441,847	115,805,837	10.45

#### Coding gene prediction

We used three independent approaches to predict coding genes: ab initio prediction, homology prediction, and prediction based on RNAseq. First, Genscan^30^, Augustus^31^, GlimmerHMM^32^, GeneID^33^, and SNAP^34^ were used for ab initio prediction, and GeMoMa^35,36^ was used to make predictions based on homologous species. Third, a reference transcript-based assembly was performed using Hisat^37^ and Stringtie^38^. Gene prediction was performed using TransDecoder v2.0 (https://github.com/TransDecoder/transdecoder.github.io) (default parameters) and GeneMarkS-T v5.1^39^ (default parameters), and PASA^40^ was used to predict unigene sequences based on transcriptome data without reference assembly. Finally, EVM^41^ was used to integrate the prediction results of the abovementioned three methods, and PASA^40^ was used to modify the prediction results. The specific forecast information is shown in Tables 6 and 7.

**Table 6:** Statistical results of gene prediction.

Method	Software	Species	Gene number
Ab initio	Genscan	—	37,606
	Augustus	—	25,530
	Gene ID	—	32,866
	SNAP	—	52,716
Homology based	GeMoMa	Anser_cygnoides	14,774
		Anas_platyrhynchos	15,228
		Gallus_gallus	15,931
		Columba_livia	15,474
RNA seq	TransDecoder	—	70,408
	GeneMarkS-T	—	32,757
	PASA	—	28,923
Integration	EVM	—	16,585

**Table 7:** Statistical table of gene information.

Gene type	Number	Length(bp)	Average length(bp)	Average number
Gene	16,585	358,694,481	2,162,764	
Exon	168,391	47,966,276	289,215	1,015
CDS	163,777	27,202,740	164,020	988
Intron	151,806	310,728,205	1,873,550	915

#### Prediction of non-coding RNA

Different strategies have been used to predict various ncRNAs, including miRNAs, rRNA, and tRNA. Based on the Rfam^42^ (default parameters) database, BLASTN was used for genome-wide comparisons to identify miRNAs and rRNA. tRNAscan-SE^43^ (default parameters) was used to identify the tRNA. Specific forecast information is presented in Table 8.

**Table 8:** Statistical results of non-coding RNA.

RNA classification	Number	Family
miRNA	205	99
rRNA	240	4
tRNA	251	23

#### Gene function annotation

**Fig. 5:**
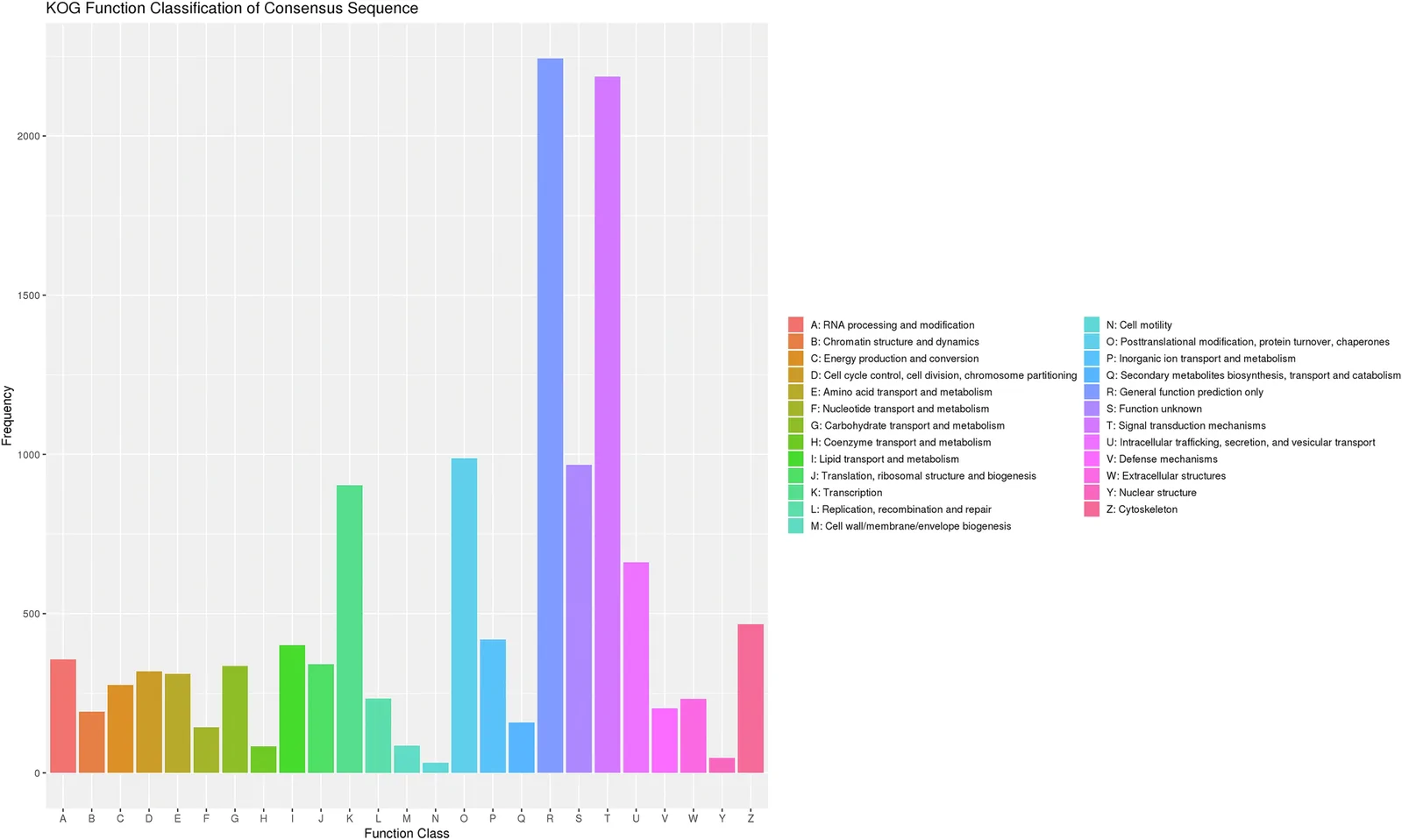
KOG features annotated categorical statistics, and.

**Fig. 6:**
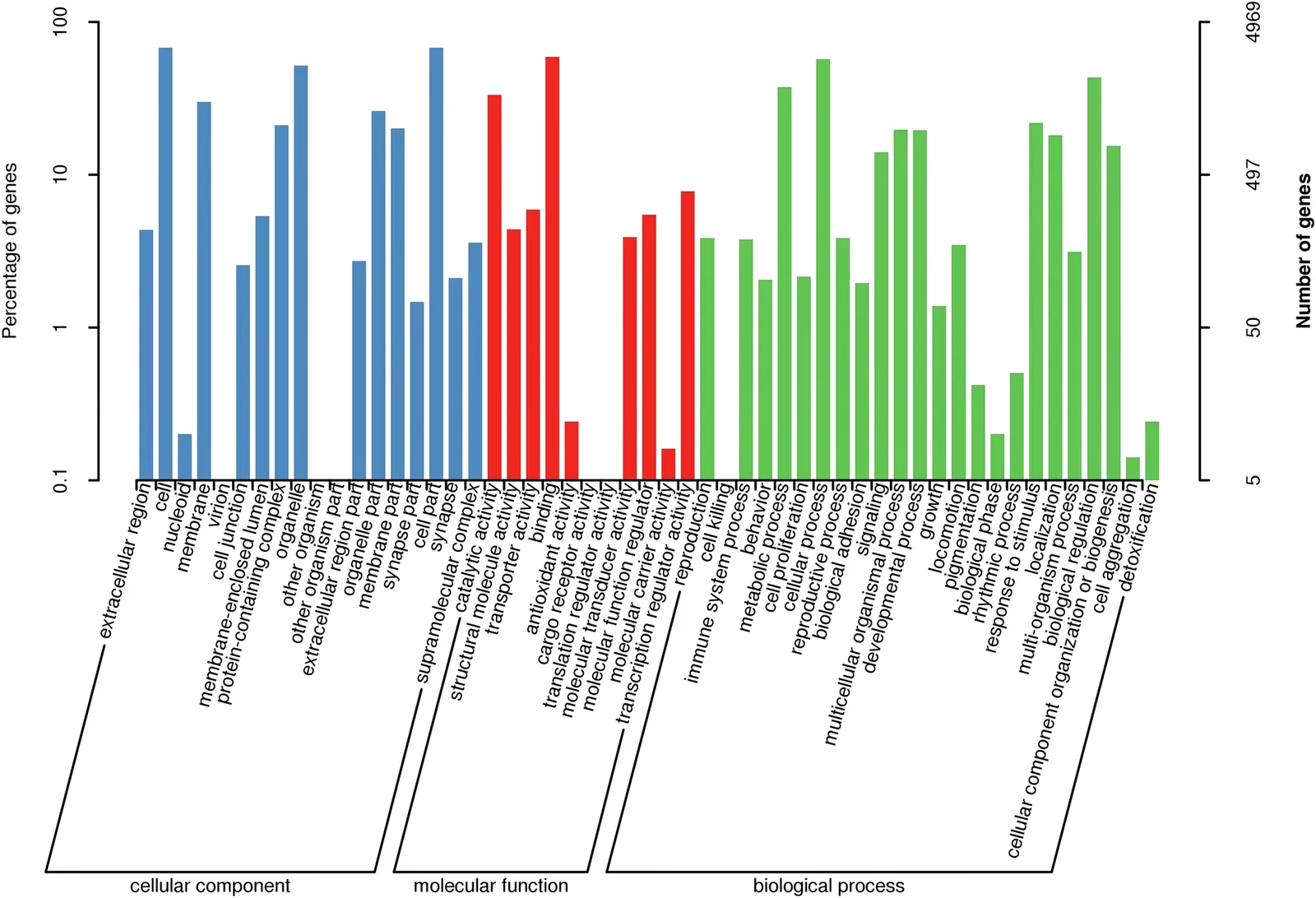
GO secondary node annotation classification statistics.

Gene functions, motifs, and protein domains were determined by comparison with public databases, including SwissProt, Non-Redundant Protein Database (NR), Kyoto Encyclopedia of Genes and Genomes (KEGG), Eukaryotic Orthologous Groups (KOG), Gene Ontology (GO), and TrEMBL. BLAST^44^ was used to compare the protein sequences against the public protein databases, and functional annotation statistics are shown in Table 9. The classification statistics shown in Figs. 5 and 6.

**Table 9:** Statistical information of gene function annotation.

Annotation database	Annotated number	Percentage(%)
GO Annotation	4,969	29.96
KEGG Annotation	10,530	63.49
KOG Annotation	11,203	67.55
TrEMBL Annotation	15,790	95.21
Nr Annotation	15,892	95.82
All Annotated	15,905	95.90

#### Pseudogene annotation

Using the predicted protein sequences, GenBlastA^45^ alignment was used to identify homologous gene sequences (possible genes) in the genome, and GeneWise^46^ was used to identify immature stop codons and frameshift mutations in gene sequences to obtain pseudogenes, as shown in Table 10.

**Table 10:** Statistics of SNPs and InDels annotation^1^.

Annotation type	UTR3	UTR5	Downstream	Exonic	Intergenic	Intronic	Splicing	Upstream	Others
SNP number	72,218.56	17,372.38	75,701.44	73,072.42	3,287,987.45	1,712,489.48	227.28	72,570.66	4,330.67
SNP percentage	1.3585%	0.3268%	1.4240%	1.3746%	61.8511%	32.2141%	0.0043%	1.3651%	0.0815%
InDels number	9,970.00	9,970.00	10,771.72	2,782.37	2,782.37	217,306.16	213.56	10,113.08	693.99
InDels percentage	1.5209%	0.3549%	1.6432%	0.4244%	61.2258%	33.1495%	0.0326%	0.0326%	0.1059%

^1^All the terms below were calculated in each pigeon breed; values represent means over individual sequences per breed.

## Technical Validation

### Quality control of sequencing results

For the 64 pigeons (6 breeds), each sample generated 6.55–9.60 Gb of raw data from each individual were obtained, with an average Q30 of 94.67% and GC content of 42.44% (Supplement Table S3 Statistics for genome alignment analysis from each individual. snp.index).

#### PacBio HiFi validation

The HiFi reads had an average accuracy of 99.8% and N50 of 12.13 kb, which enabled resolving complex genomic regions (e.g., 14.76% repetitive sequences identified via k-mer analysis). Canu assembly using these reads resulted in a contig N50 of 7.49 Mb, verifying assembly continuity.

#### Hi-C validation

Hi-C raw data (Q30 = 93.8%) were mapped to the draft assembly with a unique mapping rate of 82.5%. Valid interaction pairs accounted for 23.3% of unique reads, and scaffolding yielded 29 superscaffolds (98.59% genome coverage) with clear chromosomal interaction signals (Hi-C heatmap, Fig. 3), confirming the accuracy of chromosome anchoring.Mapping to the ZA_WK_1 genome yielded an average mapping rate of 99.23% and coverage of 99.12%, confirming the reliability of population genetic data.

### Filtration of SNP and InDel datasets

**Fig. 7:**
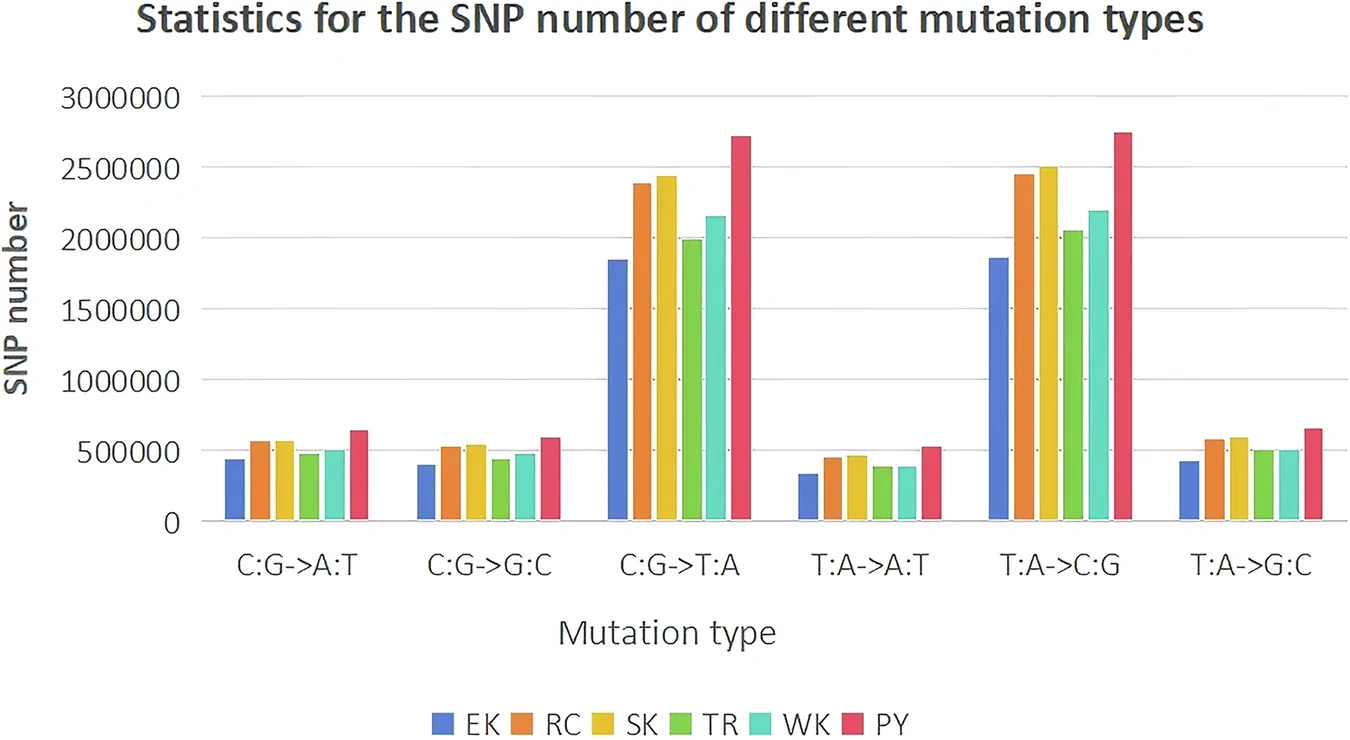
Statistics for the SNP number of different mutation types.

The joint calling strategy was used in this procedure, and over 2.27 million raw SNPs and 0.25 million raw InDels were identified in the population of six pigeon breeds.To exclude low-quality variants,we used the VariantFiltration function in GATK^47^ (Supplement Table S4 Statistics for snp and indel).The specific standards used are described in the Methods section above. After the first round of quality control, the SNPs and InDels were further filtered using VCFtools v0.1.13^48^, and only biallelic variants with a minor allele frequency of 0.01 and a minimum sequencing depth of 3 were retained. We calculated statistics for SNP types, and T:A > C:G-type mutations were mainly identified in this population (Fig. 7, Supplement Table S5).

### Summary statistics of SNPs and InDels across the whole genome

**Fig. 8:**
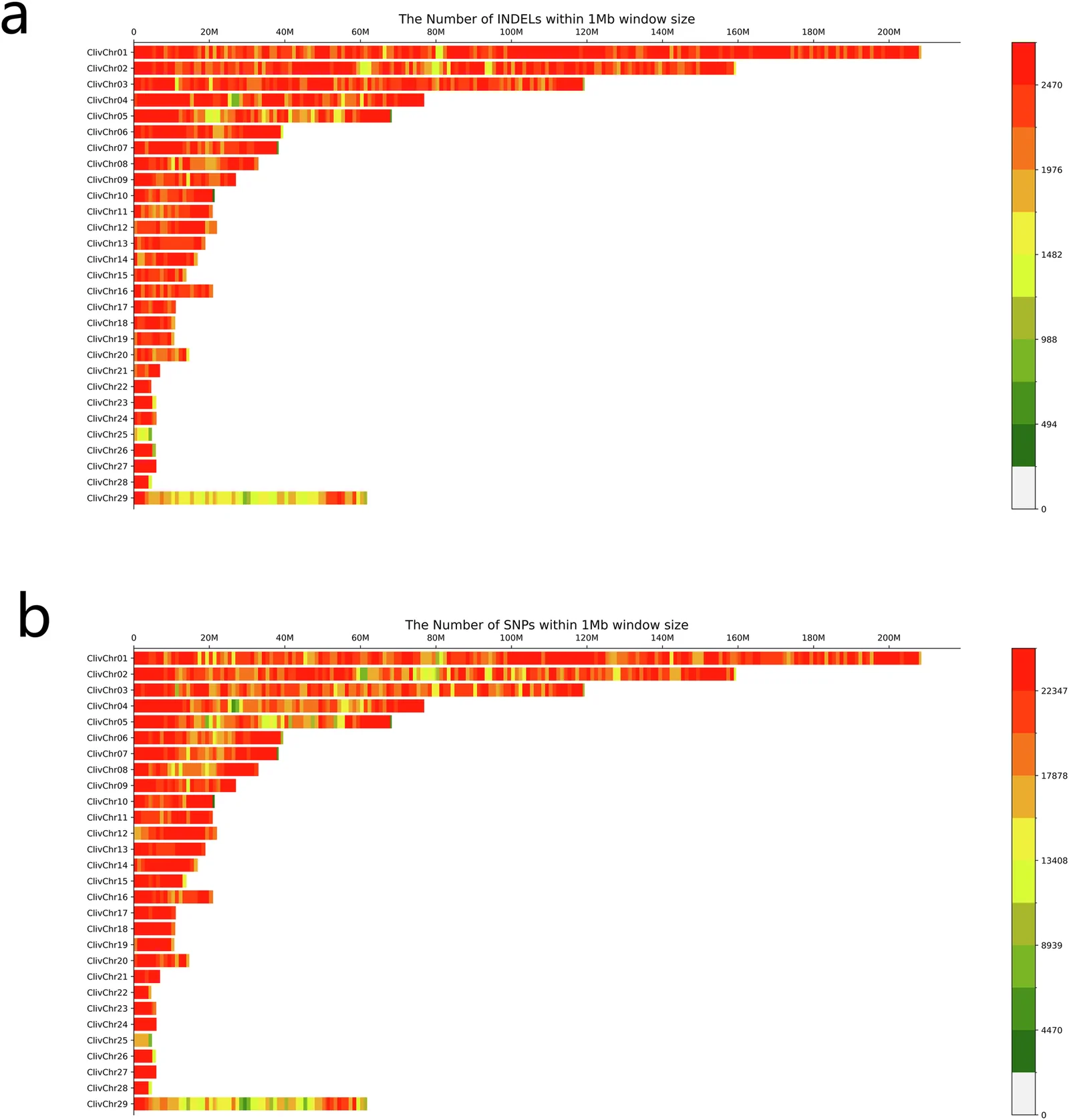
Distribution of SNP and InDel across the whole-genome of six pigeons breeds. (**a**) SNP density statistics across the whole-genome. (**b**) InDel density statistics across the whole-genome show the high density of SNPs across the whole genome.

Based on variants annotation, SNPs/InDels were mainly distributed in intronic and intergenic regions. And more SNPs rather than InDels were located in exons (Table 10, Fig. 8).

### Quality assessment of the genome assembly

In the present research work, a high-quality chromosome-scale genome assembly of the White King Pigeon was constructed by combining Hifi sequencing, short reads sequencing, and chromosome conformation capture (Hi-C) anchoring, which resulted in a genome approximately 1.11 Gb in length with contig 77.02 mb and scaffold N50  7.49 Mb, respectively. Contigs were scaffolded into 29 superscaffolds, accounting for 98.59% of the total genome size. The length of the sequence that can determine the order and direction is 1.06 Gb, accounting for 96.8% of the genome size. As shown in the Hi-C heatmap (Fig. 3), the 29 superscaffolds in the Silver King Pigeon genome could be distinguished and perfectly represented by 29 chromosomes.

To evaluate the completeness of our assembly, we carried out BUSCO (Seppey *et al*., 2019) (Benchmarking Universal Single Copy Orthologs) and CEGMA47^49^ (Core Eukaryotic Gene Mapping Approach) analyses. BUSCO results indicated that 4595 (93.49%) of the 4,915 conserved single-copy genes were present in our assembly, of which 4538 (92.33%) were single, 57 (1.16%) were duplicated, and 205 (4.17%) fragmented matches (Table 1 BUSCO assessment results). CEGMA results indicated 227 (91.53%) core genes of the 248 core eukaryotic genes were present in our assembly, It shows that the core gene in the genome is relatively complete.

### Gene prediction and annotation validation

Three independent approaches, namely ab initio prediction, homology search, and reference-guided transcriptome assembly, were used for gene prediction in a repeat-masked genome. EVM^41^ software was used to integrate the gene prediction results and generate a consensus gene set. In addition, the functional annotation of these predicted genes revealed that 95.90% (15, 905 genes) could be assigned to at least one functional term. These findings strongly suggest that the annotated gene set of the WK genome was complete.

## Data Records

Whole-genome resequencing data of pigeons were deposited in the Genome Sequence Archive (GSA) of the China National Center for Bioinformation (CNCB) under BioProject accession number PRJCA025398 and Submission ID CRA016508^50^. The PacBio HiFi reads, Illumina short paired-end reads and Hi-C sequencing reads of the White King pigeon were submitted to the GSA of CNCB Submission IDs CRA016705^51^, CRA016762^52^ and CRA016507^53^, respectively. The chromosome-level genome assembly of the White King pigeon was deposited in the NCBI GenBank under the accession number JBEHWU000000000.1^54^, with the assembly version ZA_WK_1 (YG_WK_1). The raw VCF files containing single nucleotide polymorphisms (SNPs) and insertions-deletions (InDels) for 64 pigeon samples were archived in the Genome Variation Map (GVM) of CNCB accession number GVM000785^55^. Detailed information of the sample IDs and corresponding CNCB project accessions is provided in Supplementary Table S2.

## Supplementary information

Supplementary Table S4

Supplementary Table S5

Supplementary Table S1

Supplementary Table S2

Supplementary Table S3

## Data Availability

All raw sequencing data and the final genome assembly generated in this study have been deposited in public domain databases under accession numbers as follows: 1. Whole-genome Resequencing dataset of pigeon: CRA016508 (China National Center for Bioinformation, https://ngdc.cncb.ac.cn/gsa/) 2. PacBio HiFi reads: CRA016705 (China National Center for Bioinformation, https://ngdc.cncb.ac.cn/gsa/) 3. Illumina short paired-end reads: CRA016762 (China National Center for Bioinformation, https://ngdc.cncb.ac.cn/gsa/) 4. Hi-C reads: CRA016507 (China National Center for Bioinformation, https://ngdc.cncb.ac.cn/gsa/) 5. White King pigeon genome assembly: JBEHWU010000000 (GenBank, NCBI, https://www.ncbi.nlm.nih.gov/) 6. Raw VCF file containing SNPs and InDels: GVM000785 (China National Center for Bioinformation, https://www.cncb.ac.cn/). All datasets are also accessible via the Figshare repository with the https://doi.org/10.6084/m9.figshare.28703618.

## Code availability

The software parameters and database used are described below.

1. Quality control and genome alignment

Trimmomatic (v0.36) code for trimming and quality control:

ILLUMINACLIP:adapters.fa:2:30:10 SLIDINGWINDOW:4:15 MINLEN:75

BWA (v0.7.17) code for mapping reads:mem -t 20 -M

2. *De novo* assembly and polishing

jellyfish (v2.3.0) code for K-mer distribution:

-m 21 -s 1500 M -t 10 -C reads.fasta

Canu (v1.7) code for assembly:

Canu minReadLength = 1500 minOverlapLength = 900 corOutCoverage = 80 correctedErrorRate = 0.040

polish (v1.23) code for polish: Default

3. Hi-C assisted genome assembly

Hi-C-Pro (v2.8.1) code for filtering and evaluating Hi-C data: -c config-hicpro.txt -i -o

LACHESIS (v1.0) code for Hi-C assembly:

CLUSTER_MIN_RE_SITES = 51;CLUSTER_MAX_LINK_DENSITY = 2;CLUSTER_NONINFORMATIVE_RATIO = 2;OcRDER_MIN_N_RES_IN_TRUN = 30;ORDER_MIN_N_RES_IN_SHREDS = 32

Juicebox (v1.11) code for Genome regulation: default

4. SNP and InDel

SAMtools (v1.12) code for sorting and file converting:

samtools sort -m 10 G -S ${individual}sam -o ${individual}sorted.bam -@ 10

Code for bam file indexing:

samtools index -b ${individual}.sorted.bam ${individual}.sorted.bai

Picard (1.9.2) code for remove duplicated reads:

java -Xms100g -Xmx200g -jar picard.jar MarkDuplicates INPUT = ${individual}.sorted.bam OUTPUT = ${individual}.rmdup.bam M = ${individual}.metrices.txt REMOVE_DUPLICATES = true CREATE_INDEX = true

GATK (v4.1.2.0) code for SNP/Indel extraction:

HaplotypeCaller -stand-call-conf 50–max-genotype-count 500

GATK (v4.1.2.0) code for SNP filtration:

Parameter:VariantFiltration;QD < 2.0 || QUAL < 30.0 || SOR > 3.0 || FS > 60.0 || MQ < 40.0 || MQRankSum < −12.5 || ReadPosRankSum < −8.0

GATK (v4.1.2.0) code for Indel filtration:

Parameter:VariantFiltration;QD < 2.0 || QUAL < 30.0 || FS > 200.0 || MQ < 40.0 || ReadPosRankSum < −20.0

ANNOVAR (v2019Oct24) code for SNP/indel annotation: Default

5. Tree analysis

FastTree (v2.1.10) code for tree analysis: -nt -gtr

6. Repeat sequence annotation

LTR_FINDER (v1.01) code for prediction of LTR: default

RepeatScout (v2.1) code for identification of repeat families: default

PASTEClassifier (v1.079) code for classifying TEs: default

RepeatMasker (v1.331) code for identify repeats across the genome: -nolow -no_is -norna -engine wublast

7. Coding gene prediction

Genscan code for ab initio prediction: default

Augustus (v2.4) code for ab initio prediction: default

GlimmerHMM (v3.0.4) code for ab initio prediction: default

GeneID (v1.4) code for ab initio prediction: default

SNAP (v2006-07-28) code for ab initio prediction: default

GeMoMa (v1.3.1) code for predictions based on homologous species: default

Hisat (v2.0.4) code for reference transcript based assembly:

–max-intronlen 20000, –min-intronlen 20

Stringtie (v1.2.3) code for reference transcript based assembly: default

TransDecoder (v2.0) code for gene prediction: default

GeneMarkS-T (v5.1) code for gene prediction: default

PASA (v2.0.2) code for predicting Unigene sequences:

-align_tools gmap, -maxIntronLen 20000

EVM (v1.1.1) code for integrating the prediction results: default

8. Prediction of non-coding RNA

Rfam database for identifying microRNA and rRNA: default

tRNAscan-SE database for identifying tRNA: default

9. Gene function annotation

BLAST (v2.2.3) code for gene sequence comparison: -evalue 1e-5

GenBlastA (v1.01) code for pseudogene comparison: -e 1e-5

GeneWise (v2.4) code for pseudogene comparison: default

No custom code was developed or used for the curation and/or validation of the datasets in this study. All data processing was performed using publicly available software with parameters specified in this section.
