# Supplementary material for: High-Quality Genome Assembly of the White King Pigeon for Genetic Reference of Chinese Market Pigeon Breeds
Source: Sci Data. 2026 Jun 18;13:1376. doi: 10.1038/s41597-026-07220-4 (PMC13631239; doi:10.1038/s41597-026-07220-4)
Supplement: Supplementary file 1 [file 41597_2026_7220_MOESM3_ESM.pdf]

Supplementary Table S1. Supplement information for 6 pigeon breeds.

Breeds	origin	Gender	obreviatic	Number	Age	Sampling position
er King Pig	America	female	EK	10	adult	jiang Province Pingyang County Aofeng pigeon industry Co
te King pig	America	female	WK	9	adult	jiang Province Pingyang County Aofeng pigeon industry Co
rnean pige	France	female	RC	10	adult	jiang Province Pingyang County Aofeng pigeon industry Co
ck Kee piga，	Guang (	female	SK	10	adult	jiang Province Pingyang County Aofeng pigeon industry Co
arim pigeoina，	Xin jia	female	TR	10	adult	jiang Province Pingyang County Aofeng pigeon industry Co
gyang pigeina,Zhe jiar		female	PY	14	adult	jiang Province Pingyang County Aofeng pigeon industry Co

., LTD

., LTD

., LTD

., LTD

., LTD

., LTD
