# Supplementary material for: High-Quality Genome Assembly of the White King Pigeon for Genetic Reference of Chinese Market Pigeon Breeds
Source: Sci Data. 2026 Jun 18;13:1376. doi: 10.1038/s41597-026-07220-4 (PMC13631239; doi:10.1038/s41597-026-07220-4)
Supplement: Supplementary file 2 [file 41597_2026_7220_MOESM4_ESM.pdf]

**Supplementary Table S2. Supplement information of pigeon name and CNCB proje**

| Description | Breeds | BioSample name |
|---|---|---|
| Sequencing data and genome variantion data | Silver King Pigeon | EK10 |
| Sequencing data and genome variantion data | Silver King Pigeon | EK1 |
| Sequencing data and genome variantion data | Silver King Pigeon | EK2 |
| Sequencing data and genome variantion data | Silver King Pigeon | EK3 |
| Sequencing data and genome variantion data | Silver King Pigeon | EK4 |
| Sequencing data and genome variantion data | Silver King Pigeon | EK5 |
| Sequencing data and genome variantion data | Silver King Pigeon | EK6 |
| Sequencing data and genome variantion data | Silver King Pigeon | EK8 |
| Sequencing data and genome variantion data | Silver King Pigeon | EK9 |
| Sequencing data and genome variantion data | Carnean,pigeon | RC10 |
| Sequencing data and genome variantion data | Carnean,pigeon | RC1 |
| Sequencing data and genome variantion data | Carnean,pigeon | RC2 |
| Sequencing data and genome variantion data | Carnean,pigeon | RC3 |
| Sequencing data and genome variantion data | Carnean,pigeon | RC4 |
| Sequencing data and genome variantion data | Carnean,pigeon | RC5 |
| Sequencing data and genome variantion data | Carnean,pigeon | RC6 |
| Sequencing data and genome variantion data | Carnean,pigeon | RC7 |
| Sequencing data and genome variantion data | Carnean,pigeon | RC8 |
| Sequencing data and genome variantion data | Carnean,pigeon | RC9 |
| Sequencing data and genome variantion data | Shack Kee pigeon | SK10 |
| Sequencing data and genome variantion data | Shack Kee pigeon | SK13 |
| Sequencing data and genome variantion data | Shack Kee pigeon | SK1 |
| Sequencing data and genome variantion data | Shack Kee pigeon | SK2 |
| Sequencing data and genome variantion data | Shack Kee pigeon | SK3 |
| Sequencing data and genome variantion data | Shack Kee pigeon | SK5 |
| Sequencing data and genome variantion data | Shack Kee pigeon | SK6 |
| Sequencing data and genome variantion data | Shack Kee pigeon | SK7 |
| Sequencing data and genome variantion data | Shack Kee pigeon | SK8 |
| Sequencing data and genome variantion data | Shack Kee pigeon | SK9 |
| Sequencing data and genome variantion data | Tarim pigeon | TR10 |
| Sequencing data and genome variantion data | Tarim pigeon | TR11 |
| Sequencing data and genome variantion data | Tarim pigeon | TR13 |
| Sequencing data and genome variantion data | Tarim pigeon | TR1 |
| Sequencing data and genome variantion data | Tarim pigeon | TR3 |
| Sequencing data and genome variantion data | Tarim pigeon | TR4 |
| Sequencing data and genome variantion data | Tarim pigeon | TR5 |
| Sequencing data and genome variantion data | Tarim pigeon | TR6 |
| Sequencing data and genome variantion data | Tarim pigeon | TR7 |
| Sequencing data and genome variantion data | Tarim pigeon | TR8 |
| Sequencing data and genome variantion data | Pingyang pigeon | Wb-10 |
| Sequencing data and genome variantion data | Pingyang pigeon | Wb-11 |
| Sequencing data and genome variantion data | Pingyang pigeon | Wb-12 |
| Sequencing data and genome variantion data | Pingyang pigeon | Wb-13 |
| Sequencing data and genome variantion data | Pingyang pigeon | Wb-14 |
| Sequencing data and genome variantion data | Pingyang pigeon | Wb-15 |
| Sequencing data and genome variantion data | Pingyang pigeon | Wb-1 |
| Sequencing data and genome variantion data | Pingyang pigeon | Wb-2 |
| Sequencing data and genome variantion data | Pingyang pigeon | Wb-3 |
| Sequencing data and genome variantion data | Pingyang pigeon | Wb-4 |
| Sequencing data and genome variantion data | Pingyang pigeon | Wb-5 |
| Sequencing data and genome variantion data | Pingyang pigeon | Wb-6 |

| | | | |
|---|---|---|---|
| Sequencing data and | genome variantion data | Pingyang pigeon | Wb-7 |
| Sequencing data and | genome variantion data | Pingyang pigeon | Wb-8 |
| Sequencing data and | genome variantion data | Pingyang pigeon | Wb-9 |
| Sequencing data and | genome variantion data | White King pigeon | WK10 |
| Sequencing data and | genome variantion data | White King pigeon | WK11 |
| Sequencing data and | genome variantion data | White King pigeon | WK12 |
| Sequencing data and | genome variantion data | White King pigeon | WK13 |
| Sequencing data and | genome variantion data | White King pigeon | WK1 |
| Sequencing data and | genome variantion data | White King pigeon | WK2 |
| Sequencing data and | genome variantion data | White King pigeon | WK3 |
| Sequencing data and | genome variantion data | White King pigeon | WK4 |
| Sequencing data and | genome variantion data | White King pigeon | WK8 |
| Sequencing data and | genome variantion data | White King pigeon | WK9 |
| Hi_C data | | White King pigeon | H1 |
| HiFi data | | White King pigeon | P2 |
| Survey for pigeon genome,short reads data for | | White King pigeon | S1 |
| Survey for pigeon genome,short reads data for | | White King pigeon | S2 |

**ct**

| Name in this ar | Project accession | Biosample accession |
|---|---|---|
| EK10 | PRJCA0253( | SAMC3725843 |
| EK1 | PRJCA0253( | SAMC3798960 |
| EK2 | PRJCA0253( | SAMC3725844 |
| EK3 | PRJCA0253( | SAMC3725845 |
| EK4 | PRJCA0253( | SAMC3725846 |
| EK5 | PRJCA0253( | SAMC3725847 |
| EK6 | PRJCA0253( | SAMC3725848 |
| EK8 | PRJCA0253( | SAMC3725849 |
| EK9 | PRJCA0253( | SAMC3725850 |
| RC10 | PRJCA0253( | SAMC3725861 |
| RC1 | PRJCA0253( | SAMC3725862 |
| RC2 | PRJCA0253( | SAMC3725863 |
| RC3 | PRJCA0253( | SAMC3725864 |
| RC4 | PRJCA0253( | SAMC3725865 |
| RC5 | PRJCA0253( | SAMC3725866 |
| RC6 | PRJCA0253( | SAMC3725867 |
| RC7 | PRJCA0253( | SAMC3725868 |
| RC8 | PRJCA0253( | SAMC3725869 |
| RC9 | PRJCA0253( | SAMC3725870 |
| SK10 | PRJCA0253( | SAMC3725871 |
| SK13 | PRJCA0253( | SAMC3725872 |
| SK1 | PRJCA0253( | SAMC3725873 |
| SK2 | PRJCA0253( | SAMC3725874 |
| SK3 | PRJCA0253( | SAMC3725875 |
| SK5 | PRJCA0253( | SAMC3725876 |
| SK6 | PRJCA0253( | SAMC3725877 |
| SK7 | PRJCA0253( | SAMC3725878 |
| SK8 | PRJCA0253( | SAMC3725879 |
| SK9 | PRJCA0253( | SAMC3725880 |
| TR10 | PRJCA0253( | SAMC3725881 |
| TR11 | PRJCA0253( | SAMC3725882 |
| TR13 | PRJCA0253( | SAMC3725883 |
| TR1 | PRJCA0253( | SAMC3725884 |
| TR3 | PRJCA0253( | SAMC3725885 |
| TR4 | PRJCA0253( | SAMC3725886 |
| TR5 | PRJCA0253( | SAMC3725887 |
| TR6 | PRJCA0253( | SAMC3725888 |
| TR7 | PRJCA0253( | SAMC3725889 |
| TR8 | PRJCA0253( | SAMC3725890 |
| PY10 | PRJCA0253( | SAMC3725906 |
| PY11 | PRJCA0253( | SAMC3725907 |
| PY12 | PRJCA0253( | SAMC3725908 |
| PY13 | PRJCA0253( | SAMC3725909 |
| PY14 | PRJCA0253( | SAMC3725910 |
| PY15 | PRJCA0253( | SAMC3725911 |
| PY1 | PRJCA0253( | SAMC3725912 |
| PY2 | PRJCA0253( | SAMC3725913 |
| PY3 | PRJCA0253( | SAMC3725914 |
| PY4 | PRJCA0253( | SAMC3725915 |
| PY5 | PRJCA0253( | SAMC3725916 |
| PY6 | PRJCA0253( | SAMC3725917 |

| | | |
|---|---|---|
| PY7 | PRJCA0253( | SAMC3725918 |
| PY8 | PRJCA0253( | SAMC3725919 |
| PY9 | PRJCA0253( | SAMC3725920 |
| WK10 | PRJCA0253( | SAMC3725936 |
| WK11 | PRJCA0253( | SAMC3725937 |
| WK12 | PRJCA0253( | SAMC3725938 |
| WK13 | PRJCA0253( | SAMC3725939 |
| WK1 | PRJCA0253( | SAMC3725940 |
| WK2 | PRJCA0253( | SAMC3725941 |
| WK3 | PRJCA0253( | SAMC3725942 |
| WK4 | PRJCA0253( | SAMC3725943 |
| WK8 | PRJCA0253( | SAMC3725944 |
| WK9 | PRJCA0253( | SAMC3725945 |
| Hi_C data | PRJCA0264( | SAMC3759594 |
| HiFi data | PRJCA0264( | SAMC3762488 |
| Illumina short pa | PRJCA0264( | SAMC3767545 |
| Illumina short pa | PRJCA0264( | SAMC3767546 |
