# Supplementary material for: High-Quality Genome Assembly of the White King Pigeon for Genetic Reference of Chinese Market Pigeon Breeds
Source: Sci Data. 2026 Jun 18;13:1376. doi: 10.1038/s41597-026-07220-4 (PMC13631239; doi:10.1038/s41597-026-07220-4)
Supplement: Supplementary file 3 [file 41597_2026_7220_MOESM5_ESM.pdf]

Supplementary Table S3. Statistics for genome alignment analysis from each individual.

Breed	Individual	Clean reads	Mapped reads	Mapped	Coverage	Depth(x)
EK	EK10	68294170.00	67740987.00	99.19	96.98	8.05
EK	EK1	53413642.00	52997015.00	99.22	95.84	6.43
EK	EK2	61179410.00	60616559.00	99.08	96.72	7.43
EK	EK3	63266516.00	62507317.00	98.80	96.99	7.79
EK	EK4	63195624.00	62393039.00	98.73	96.46	7.69
EK	EK5	71169682.00	70749780.00	99.41	98.79	9.27
EK	EK6	61951668.00	61233028.00	98.84	97.10	7.52
EK	EK8	73657262.00	73068003.00	99.20	97.47	8.92
EK	EK9	73326408.00	72754462.00	99.22	97.87	8.73
RC	RC10	114326638.00	113594947.00	99.36	98.26	14.94
RC	RC1	202952694.00	200923167.00	99.00	99.46	24.61
RC	RC2	69092696.00	68387950.00	98.98	97.09	8.36
RC	RC3	71353238.00	70589758.00	98.93	96.94	8.60
RC	RC4	91538694.00	90312075.00	98.66	97.91	11.07
RC	RC5	80471000.00	79972079.00	99.38	98.04	9.90
RC	RC6	95132248.00	94542428.00	99.38	98.78	12.23
RC	RC7	80437934.00	79641598.00	99.01	97.72	10.15
RC	RC8	87777266.00	87189158.00	99.33	98.21	10.81
RC	RC9	66979722.00	66457280.00	99.22	96.40	8.31
SK	SK10	75604692.00	75151063.00	99.40	98.91	9.71
SK	SK13	79028132.00	78261559.00	99.03	95.75	9.99
SK	SK1	201136092.00	199828707.00	99.35	98.34	23.14
SK	SK2	67875404.00	67393488.00	99.29	97.88	8.47
SK	SK3	70628918.00	70141578.00	99.31	97.94	9.21
SK	SK5	81479080.00	80949465.00	99.35	99.11	10.23
SK	SK6	79630944.00	79089453.00	99.32	98.20	10.12
SK	SK7	69294412.00	68594538.00	98.99	97.20	8.66
SK	SK8	89036694.00	88511377.00	99.41	99.18	11.25
SK	SK9	98801474.00	97912260.00	99.10	98.37	12.39
TR	TR10	76210024.00	75668932.00	99.29	82.82	9.64
TR	TR11	75774812.00	75282275.00	99.35	83.95	9.57
TR	TR13	222521394.00	221408787.00	99.50	90.17	27.76
TR	TR1	75406128.00	75074341.00	99.56	71.74	9.66
TR	TR3	75719202.00	74901434.00	98.92	98.98	9.69
TR	TR4	74283798.00	73139827.00	98.46	98.13	9.37
TR	TR5	74364214.00	73724681.00	99.14	98.97	9.20
TR	TR6	77442654.00	77039952.00	99.48	62.02	9.88
TR	TR7	87954776.00	87506206.00	99.49	83.16	11.32
TR	TR8	79325784.00	78952952.00	99.53	84.48	10.12
PY	PY10	79083156.00	78284416.00	98.99	98.30	10.40
PY	PY11	84440688.00	83511840.00	98.90	98.27	11.08
PY	PY12	93651866.00	92743442.00	99.03	98.36	12.30
PY	PY13	82413826.00	81573204.00	98.98	98.32	10.86
PY	PY14	75261452.00	74621729.00	99.15	99.18	9.90
PY	PY15	89129566.00	88389790.00	99.17	99.32	11.74
PY	PY1	104515718.00	103690043.00	99.21	99.41	13.73
PY	PY2	82333692.00	81535055.00	99.03	99.24	10.83
PY	PY3	93805864.00	92877185.00	99.01	98.49	12.34
PY	PY4	88882076.00	88011031.00	99.02	98.37	11.68
PY	PY5	76325326.00	75691825.00	99.17	99.23	10.05
PY	PY6	78962172.00	78085691.00	98.89	98.22	10.38
PY	PY7	70292476.00	69561434.00	98.96	98.18	9.27
PY	PY8	79298356.00	78449863.00	98.93	99.06	10.41

PY	PY9	72693068.00	71987945.00	99.03	98.22	9.60
WK	WK10	73269096.00	72470462.00	98.91	97.43	9.02
WK	WK11	74963568.00	74004034.00	98.72	97.46	9.10
WK	WK12	78170828.00	77428205.00	99.05	97.62	9.62
WK	WK13	76265924.00	75762568.00	99.34	98.38	9.57
WK	WK1	232525834.00	230084312.00	98.95	98.57	27.53
WK	WK2	81388100.00	80500969.00	98.91	97.60	9.87
WK	WK3	91511596.00	90413456.00	98.80	98.64	11.32
WK	WK4	63017784.00	62349795.00	98.94	96.79	7.78
WK	WK8	66736908.00	66056191.00	98.98	96.63	8.15
WK	WK9	61898242.00	61074995.00	98.67	96.54	7.64
